# Anesthesia inhibited corticospinal excitability and attenuated the modulation of repetitive transcranial magnetic stimulation

**DOI:** 10.1186/s12871-022-01655-z

**Published:** 2022-04-19

**Authors:** Xin Wang, Tengfei Wang, Jingna Jin, He Wang, Ying Li, Zhipeng Liu, Tao Yin

**Affiliations:** 1 Institute of Biomedical Engineering, Chinese Academy of Medical Sciences & Peking Union Medical College, Tianjin, China; 2 Neuroscience Center, Chinese Academy of Medical Sciences, Beijing, China

**Keywords:** Transcranial magnetic stimulation (TMS), Motor evoked potential (MEP), Anesthesia

## Abstract

**Background:**

Lots of studies have measured motor evoked potential (MEP) induced by transcranial magnetic stimulation (TMS) in anesthetized animals. However, in awake animals, the measurement of TMS-induced MEP is scarce as lack of sufficient restraint. So far, the explicit study of anesthesia effects on corticospinal excitability and repetitive TMS (rTMS) induced modulation is still lacking. This study aimed to: (1) measure TMS-induced MEP in both awake restrained and anesthetized rats, (2) investigate the effect of anesthesia on corticospinal excitability, and (3) on rTMS-induced modulation.

**Methods:**

MEP of eighteen rats were measured under both wakefulness and anesthesia using flexible binding and surface electrodes. Peak-to-peak MEP amplitudes, resting motor threshold (RMT) and the slope of stimulus response (SR) were extracted to investigate anesthesia effects on corticospinal excitability. Thereafter, 5 or 10 Hz rTMS was applied with 600 pulses, and the increase in MEP amplitude and the decrease in RMT were used to quantify rTMS-induced modulation.

**Results:**

The RMT in the awake condition was 44.6 ± 1.2% maximum output (MO), the peak-to-peak MEP amplitude was 404.6 ± 48.8 μV at 60% MO. Under anesthesia, higher RMT (55.6 ± 2.9% MO), lower peak-to-peak MEP amplitudes (258.6 ± 32.7 μV) and lower slope of SR indicated that the corticospinal excitability was suppressed. Moreover, under anesthesia, high-frequency rTMS still showed significant modulation of corticospinal excitability, but the modulation of MEP peak-to-peak amplitudes was weaker than that under wakefulness.

**Conclusions:**

This study measured TMS-induced MEP in both awake and anesthetized rats, and provided explicit evidence for the inhibitory effects of anesthesia on corticospinal excitability and on high-frequency rTMS-induced modulation of MEP.

## Background

Transcranial magnetic stimulation (TMS), a noninvasive brain stimulation technique that induces an electric current in the brain through a varying magnetic field, has been widely used in clinical and scientific applications [1, 2]. TMS applied to the motor cortex allows for stimulation of the corticospinal tract and eliciting motor evoked potential (MEP) in peripheral muscles [3]. When induced MEP reaches a threshold amplitude at rest, the corresponding machine output is determined as resting motor threshold (RMT) [4, 5]. Both MEP and RMT can be used as markers of corticospinal excitability as well as the modulation of excitability by repetitive TMS (rTMS) [6, 7].

In human TMS studies, MEP is commonly measured via surface electrodes in awake states [8]. In awake animals, MEP measurement entails practical difficulties due to the lack of sufficient restraint. So far, only one study measured TMS-induced MEP in the awake condition roughly [9]. In most animal experiments, MEP was not measured [10–12]. In some studies, MEP was measured under anesthesia by inserting electrodes [3, 6, 7, 13–18], which provided an objective basis for judging the RMT and quantifying the corticospinal excitability. However, it is not known whether the corticospinal excitability was affected by anesthesia.

Several studies have confirmed that anesthetics impacted neural activities, including significant downregulation of Cxcl12 mRNA expression in primary hippocampal neurons[19], suppressing neural activities in cerebral cortex [20], inhibiting excitatory transmitter release by blocking presynaptic Ca^2+^ channels or extracellular mechanisms [21], and leading to distinct spatiotemporal activity in principal neurons of the mouse olfactory cortex [22]. Not only that, a preliminary study also showed that the modulation of functional connectivity by low-frequency rTMS varied considerably among the isoflurane, propofol and dexmedetomidine groups [23]. Surprisingly, high-frequency rTMS increased BDNF and GluR1 in awake animals while decreasing them in isoflurane-anesthetized animals [11]. Thus, we speculated that anesthesia might also have effects on MEP and rTMS-induced modulation of MEP. Studies on patients showed that MEP was inhibited with desflurane, sevoflurane or propofol in a dose-dependent manner [24, 25]. However, two studies on rats revealed no differences in the mean MEP amplitude under different anesthesia [14, 16]. There is still insufficient evidence to determine whether anesthesia affects MEP and rTMS-induced modulation in rats.

In our opinion, to investigate the influence of anesthesia on corticospinal excitability and rTMS-induced modulation, an explicit comparison study between anesthetized and awake animals is very necessary, which is currently most lacking. In a study by Linden et al., a light cloth stockinet was used to confine the awake rat on a wooden board, and TMS-induced MEP was measured, but Linden et al. did not measure MEP in anesthetized rats under the same condition [9]. In another study by Hsieh et al., a platform with 4 straps was used to restrict the awake rat, and TMS-induced mechanomyogram was measured, again confirming the feasibility of flexible bindings [15]. However, the study did not measure TMS-induced MEP in unanesthetized rats. In the study of Cermak et al., a headpost was implanted into rat skull to guide the TMS target and RMT was estimated by observing limb/paw twitches, but MEP was not measured [26]. One study by Gersner et al. demonstrated that propofol modulated MEP in a dose-dependent manner, but it was not clear whether this was an explicit comparison between awake and anesthetized rats [27].

In this study, we aimed to construct explicit comparisons between awake and anesthetized rats. The effects of anesthesia on corticospinal excitability were investigated by fully measuring and comparing MEP, RMT and stimulus response (SR) during wakefulness and anesthesia. The effects of anesthesia on rTMS-induced modulation were investigated by fully measuring and comparing high frequency rTMS-induced increase of MEP and decrease of RMT during wakefulness and anesthesia. We hypothesized that anesthesia would inhibit corticospinal excitability and rTMS-induced modulation. This study would reveal explicit evidence for the influence of anesthesia on corticospinal excitability and provide reference for rTMS applied under anesthesia.

## Methods

### Animal preparation and grouping

Eighteen adult male Sprague–Dawley rats (300–350 g) were obtained from HFK Bioscience Co., Ltd. (Beijing, China). The rats were housed in group of 3, with soft sawdust in cages. The room temperature was 24 ± 2 °C, lights were on at 7:00 a.m. with a 12 h light/dark cycle, and water and food were available ad libitum. All experiments were approved by the Ethics Committee of the Institute of Biomedical Engineering, Chinese Academy of Medical Sciences& Peking Union Medical College (Approval No. IRM-DWLL-2019115). All operations were in accordance with the ARRIVE guidelines 2.0 (Animal Research: Reporting of In Vivo Experiments) [28].

**Fig. 1:**
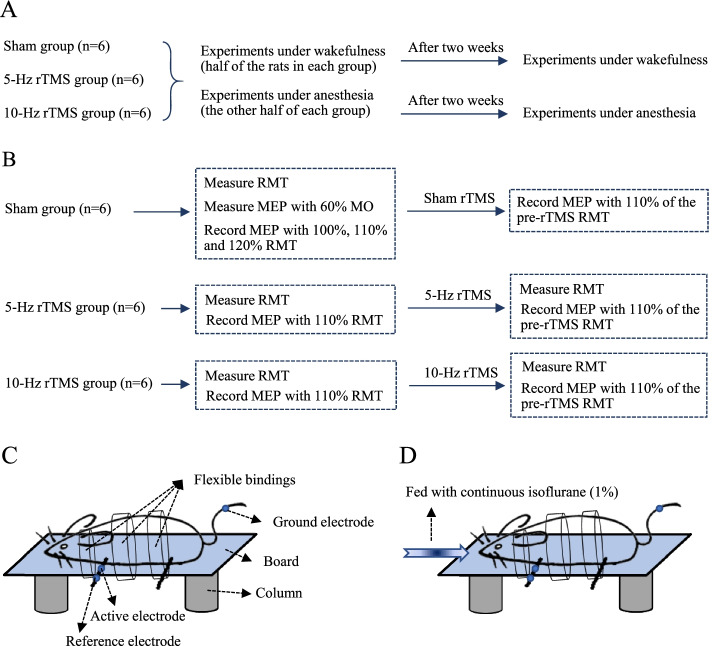
Procedure and platform for experiments under wakefulness and anesthesia. **A** Overall procedure. **B** Process for each experiment **C** Platform for awake rats **D** Platform for anesthetized rats

The rats were randomly divided into three groups according to the method of random number table: sham group (n = 6), 5-Hz rTMS group (n = 6) and 10-Hz rTMS group (n = 6). In each group, two experiments were performed, one under wakefulness and the other under anesthesia. The order of these two experiments was balanced and random. The interval between experiments under wakefulness and anesthesia was 2 weeks. The overall procedure for experiments under wakefulness and anesthesia was shown in Fig. 1A and the process for each experiment was shown in Fig. 1B. After the last treatment and data collection, the rats were euthanized by intraperitoneal injection of sodium pentobarbital at 200 mg/kg.

### Platform for fixing rats

Each rat was placed on a board, belly down. The board was supported by two columns that allowed the rats' limbs to hang down. A customized small animal anesthesia machine (R520; RWD Life Science Co., Ltd., Shenzhen, China) with a mixture of medical oxygen and isoflurane (4%) was used to temporarily anesthetize rats in an induction box. For the wakefulness experiments, three flexible bindings were used to confine the rat’s neck and back to the board to ensure significant restriction, and the isoflurane was removed immediately after the animal was securely fixed. For the anesthesia experiments, the isoflurane (1%) was continuously fed through the respiratory face mask [11]. The rats were allowed to breath spontaneously without assistance. The platforms for awake and anesthetized rats were shown in Fig. 1C and 1D.

Before the experiment, three stages of acclimation were performed. In the first stage, the rats adapted to the living environment for two days. In the second stage, acclimation of the restriction training was performed once a day for three days. In the third stage, the acclimation of the noise of TMS was performed for two days. During the experiment, the rats were in good physiological condition and showed no discomfort.

### RMT and MEP measurements

RMT and MEP were measured based on EMG recordings. After the rat was fixed, the left forelimb was depilated and the brachioradialis was located via palpation. The EMG was recorded via Ag/AgCl surface electrodes in a belly-tendon montage (Micromed, Mogliano Veneto, Italy) for both awake and anesthetized rats, with the bandpass filtering of 0.1–1000 Hz and the sampling rate of 32,768 Hz. A ground electrode was attached onto the tail. A small amount of conductive gel was applied between the electrodes and the muscles to reduce skin resistance.

A Magstim Rapid^2^ stimulator (Magstim Co. Ltd., Whitland, UK) was used to generate single-pulse TMS. A figure-of-eight coil (Magstim Co. Ltd., Whiteland, UK) with an internal diameter of 8 mm and an outer diameter of 30 mm was fixed 2 mm from the head with a holder to minimize the pressure on the rat’s head. Viewed from the top, the direction of coil current was clockwise in the left circular and counterclockwise in the right circular. Based on the pre-determined induced electric field, the center of the coil was located over the right motor cortex and moved craniocaudally and mediolaterally to identify the “hot-spot” (often 0.5 cm lateral to bregma) [3, 29]. RMT was defined as the lowest stimulus intensity that elicited MEP in the contralateral brachioradialis with a peak-to-peak amplitude above 50 μV in at least 5 out of 10 trails. The inter-pulse interval was 7 s [7, 15] and the step size of stimulatus intensity was 1% maximum output (MO, 1.2 T). Due to signal noise and the polymorphic nature of the MEP we often observed MEP amplitudes of ≥ 100 μV in real time [23, 30, 31].

For the sham group, RMT and the MEP with 60% MO were measured under both wakefulness and anesthesia. Then, MEP with the stimulus intensity of 100%, 110% and 120% RMT were recorded respectively. For 5-Hz and 10-Hz groups, RMT was measured and MEP with the stimulus intensity of 110% RMT was recorded under wakefulness and anesthesia.

### rTMS

After the initial RMT and MEP measurements, high-frequency rTMS was applied for both awake and anesthetized rats. For the 5-Hz and 10-Hz rTMS groups, the stimulus frequencies of rTMS were 5 and 10 Hz respectively [32, 33]. The session consisted of 600 pulses with the pulse width of 280 μs [34]. The coil was positioned in a manner consistent with that during initial RMT and MEP measurements. For the sham rTMS group, the frequency of rTMS was 5 Hz, and the coil was placed 8 cm away from the head so as not to subject the rat to magnetic stimulation.

After rTMS, the RMT was remeasured in the 5-Hz and 10-Hz rTMS groups. In the sham group, the RMT was not remeasured again, as it would be essentially unchanged over such a short time interval in the absence of effective stimulation. After rTMS, MEP with the stimulus intensity of 110% of the pre-rTMS RMT was recorded, in all three groups. Thus, the comparison of MEP before and after rTMS was made under the same intensity. The increase in MEP amplitude and decrease in RMT were calculated to evaluate the modulation of corticospinal excitability induced by high-frequency rTMS. The rTMS-induced modulation was compared between wakefulness and anesthesia experiments.

### Data processing and statistical analysis

The RMT, peak-to-peak amplitude of MEP and the slope of SR were calculated to quantify corticospinal excitability and were used to investigate anesthesia effects on the corticospinal excitability. The peak-to-peak amplitudes of MEP were calculated using MATLAB (version 2018). First, the EMG recordings were preprocessed, namely, 50-Hz power interference and baseline drift were removed using inverse Fourier transform. Second, the EMG recordings under the same condition were segmented by TMS pulses and averaged. As the appearance of MEP was fixed in time relative to the TMS pulse, the superposition average allowed the extraction of the MEP. Then, the peak-to-peak amplitudes of MEP were calculated. The SR with increasing stimulus intensity was quantified by the polynomial linear fitting. The slope of SR was calculated based on the peak-to-peak amplitudes of 120% RMT and the peak-to-peak amplitudes of 100% RMT.

To quantify the rTMS-induced modulation, delta RMT was calculated by subtracting the RMT before rTMS from the RMT after rTMS, and delta MEP was calculated by subtracting the peak-to-peak amplitudes of MEP before rTMS from that after rTMS. Delta RMT and delta MEP were compared between wakefulness and anesthesia to investigate the effects of anesthesia on rTMS- induced modulation.

The data were expressed as mean ± standard error of the mean (SEM). Paired *t* tests were used to compare MEP and RMT during wakefulness and anesthesia. Two-way or multi-factor analysis of variance (ANOVA) was used to assess the effects of anesthesia, stimulus intensity, and stimulus frequency (5 or 10 Hz) on MEP properties and rTMS-induced modulation, followed by post-hoc Bonferroni tests. Shapiro–Wilk normality test was performed to determine the normal distribution, N (0, 1). All statistical analyses were performed using SPSS software (version 21.0) and the significance level was set at 0.05.

## Results

### RMT and MEP under wakefulness and anesthesia

**Fig. 2:**
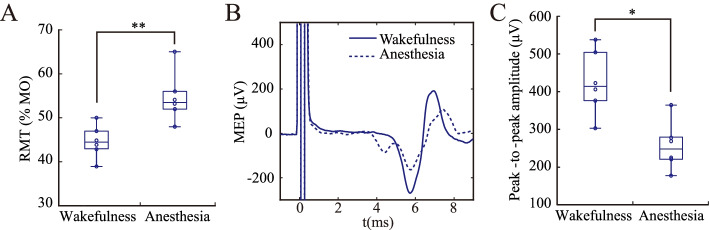
Resting motor threshold RMT and Motor evoked potentials MEP under wakefulness and anesthesia. **A** RMT. **B** MEP **C** Peak-to-peak amplitude of MEP. Anesthesia increased RMT and decreased the amplitude of MEP. The data were expressed as mean ± standard error of the mean (SEM). *P < 0.05, **P < 0.01. The data satisfies the normality

The RMT and the MEP at 60% MO from the sham group were shown in Fig. 2. As shown in Fig. 2A, the RMT under wakefulness was 44.6 ± 1.2% MO. Under anesthesia, the RMT was 55.6 ± 2.9% MO, an increase of 11.0% MO compared to that under wakefulness. There was a significant difference in RMT under the two states according to the paired-samples *t* test (Fig. 2A, *P* = 0.002).

As shown in Fig. 2B, “0” represented the time point of the stimulation, and the baseline prior to stimulation was stable. MEP appeared at 5–8 ms after stimulation and mainly consisted of a negative peak and a positive peak. With the stimulus intensity of 60% MO, the peak-to-peak amplitude of MEP was 404.6 ± 48.8 μV under wakefulness and 258.6 ± 32.7 μV under anesthesia. The peak-to-peak MEP amplitude under anesthesia was significantly different from that under wakefulness according to the paired-samples *t* test (Fig. 2C, *P* = 0.024).

Both the increase in RMT and the decrease in peak-to-peak MEP amplitude reflected the inhibitory effect of anesthesia on corticospinal excitability.

### MEP induced by increasing stimulus intensities

**Fig. 3:**
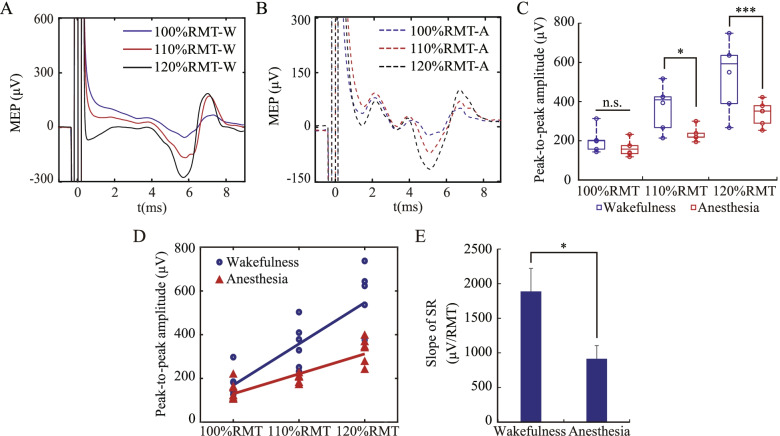
Motor evoked potential (MEP) induced by increasing stimulus intensities. **A** MEP under wakefulness **B** MEP under anesthesia. “W” (solid line) denotes Wakefulness and “A” (dashed line) denotes Anesthesia **C** Peak-to-peak MEP amplitudes **D** SR based on linear fitting. **E** The slope of SR. Anesthesia inhibited the stimulus response of TMS. The data were expressed as mean ± standard error of the mean (SEM). *P < 0.05, ***P < 0.001, n.s.: no significance. The data satisfies the normality

Based on the measured RMT, the stimulus intensities were set as 100%, 110%, and 120% RMT respectively in the sham group. The corresponding MEP signals were recorded, under both wakefulness and anesthesia, as shown in Fig. 3A and 3B. According to two-way repeated-measures ANOVA, there were significant differences among the three stimulus intensities (F(2, 35) = 28.08, *P* < 0.001), and between wakefulness and anesthesia (F(1, 35) = 17.25, *P* < 0.001). More precisely, the peak-to-peak MEP amplitudes significantly increased with increasing stimulus intensity (*P* < 0.001 for wakefulness, *P* = 0.003 for anesthesia) and, at the intensities of 110% and 120% RMT (but not 100% RMT), the peak-to-peak MEP amplitudes were significantly lower under anesthesia than under wakefulness (Fig. 3C, 110% RMT, *P* = 0.013; 120% RMT, *P* < 0.001).

The interaction effect results (F(2, 35) = 2.70, *P* = 0.079) suggested a trend that anesthesia may affect the increase in peak-to-peak MEP amplitudes induced by the increasing stimulus intensity. The SR with increasing stimulus intensity was quantified by linear fitting, as shown in Fig. 3D. The SR slope reflected that the degree of corticospinal excitability increased with the increase of stimulus intensity. As shown in Fig. 3E, the SR slope under anesthesia was significantly lower than that under wakefulness (*P* = 0.023), reflecting the inhibitory effect of anesthesia on corticospinal excitability.

### rTMS-induced modulation of RMT

**Fig. 4:**
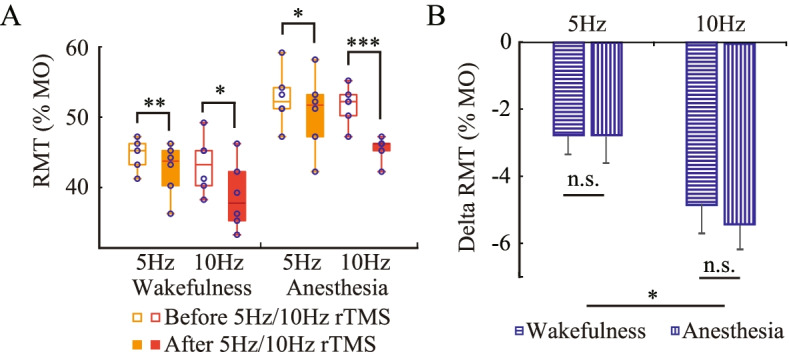
Repetitive transcranial magnetic stimulation (rTMS) -induced modulation of resting motor threshold (RMT). **A** RMT. **B** Decrease in RMT. Anesthesia did not affect the rTMS-induced modulation of RMT. The data were expressed as mean ± standard error of the mean (SEM). *P < 0.05, **P < 0.01, ***P < 0.001, n.s.: no significance. The data satisfies the normality

Figure 4A showed the RMT before and after both 5 and 10 Hz rTMS. For the sham rTMS, the RMT was not remeasured, as it would be essentially unchanged over such a short time interval in the absence of effective stimulation. The RMT after rTMS was significantly lower than that before rTMS according to the multi-factor ANOVA (F(1, 47) = 39.997, *P* < 0.001), which reflected the facilitation of corticospinal excitability by rTMS.

Under both wakefulness and anesthesia, the rTMS-induced decrease in RMT was calculated and compared, to explore the effects of anesthesia on rTMS-induced modulation of RMT. As shown in Fig. 4B, the decrease in RMT induced by 10 Hz rTMS was significantly greater than the decrease induced by 5 Hz rTMS (F(1, 23) = 5.911, *P* = 0.022). However, the decrease in RMT between wakefulness and anesthesia was not significantly different (F(1, 23) = 0.065, *P* = 0.800), indicating that anesthesia did not affect the rTMS-induced modulation of RMT.

### rTMS-induced modulation of MEP

**Fig. 5:**
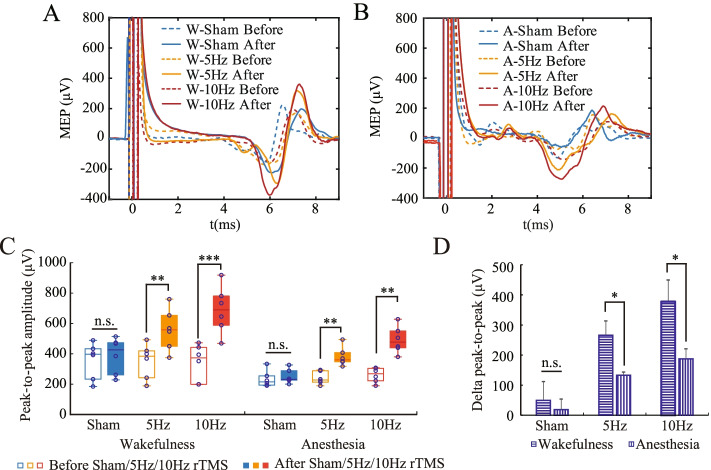
Repetitive transcranial magnetic stimulation (rTMS) -induced modulation of Motor evoked potentials (MEP). **A** MEP under wakefulness **B** MEP under anesthesia. “W” denotes Wakefulness and “A” denotes Anesthesia. Dashed lines denote MEP before rTMS and solid lines denote MEP after MEP **C** Peak-to-peak MEP amplitudes **D** Increase in peak-to-peak MEP amplitudes. Anesthesia inhibited the rTMS-induced modulation of MEP. The data were expressed as mean ± standard error of the mean (SEM). *P < 0.05, **P < 0.01, ***P < 0.001, n.s.: no significance. The data satisfies the normality

MEP before and after rTMS from all three groups were shown in Fig. 5A and 5B, respectively under wakefulness and anesthesia. The MEP was recorded at a stimulus intensity of 110% RMT. According to multi-factor ANOVA, there were significant differences before and after rTMS (F(1, 71) = 27.736, *P* < 0.001), among the three groups (F(2, 71) = 6.194, *P* = 0.003). The post-hocs showed that the peak-to-peak amplitudes after rTMS were significantly higher than that before rTMS in 5-Hz rTMS group (*p* = 0.001) and 10-Hz rTMS group (*p* < 0.001), as shown in Fig. 5C. For the sham group, there was no significant difference in MEP before and after rTMS (*p* = 0.532).

Under both wakefulness and anesthesia, the increase in peak-to-peak MEP amplitudes was calculated and compared, to explore the effects of anesthesia on rTMS-induced modulation of MEP. As shown in Fig. 5D, the increase in peak-to-peak MEP amplitudes induced by 10-Hz rTMS was greater than that induced by 5-Hz rTMS (F(1, 23) = 12.111, *P* < 0.001). Importantly, the increase in peak-to-peak MEP amplitude induced by rTMS was significantly lower under anesthesia than under wakefulness (F(1, 23) = 7.800, *P* = 0.008), which reflected the inhibitory effect of anesthesia on the rTMS-induced modulation of MEP.

## Discussion

Due to the practical difficulties while shackling awake rats, the meticulous measurement of TMS-induced MEP in awake rats is not easy. As a result, the explicit comparison of TMS-induced MEP and rTMS-induced modulation between anesthetized and awake rats is very lacking. In this study, flexible bindings were used to confine awake rats on the board, and surface electrodes were used to measure TMS-induced MEP in awake rats. More importantly, explicit comparisons between awake and anesthetized rats were constructed. Our results showed that anesthesia inhibited the corticospinal excitability, represented as higher RMT, lower peak-to-peak amplitudes of MEP and lower SR slope, compared to that under wakefulness. It's worth noting that under anesthesia, high-frequency rTMS still showed significant modulation of corticospinal excitability, but the modulation of MEP peak-to-peak amplitudes was weaker than that under wakefulness.

### RMT and MEP measurement in awake rats

In this study, the RMT under wakefulness was 44.6 ± 1.2% MO (Fig. 2A) and MEP with 60% MO was 404.6 ± 48.8 μV (Fig. 2C). In the study by Linden et al., the mean RMT in awake rats was 31 ± 7.4% MO and the peak-to-peak MEP amplitude of 100% RMT was more than 4.6 ± 2.72 mV [9]. As the magnetic stimulator used in our study (Rapid^2^; Magstim Co. Ltd., Whitland, UK) was different from that used in the study by Linden et al. (model MS-10; Caldwell Laboratories, Inc., Kennewick, WA, USA), the MO is different, so direct comparisons of RMT and MEP (% MO) are inappropriate. However, in terms of measurement methods, this study is more detailed than the study by Linden et al. [9]. In our methods, surface electrodes were used instead of needle electrodes to measure MEP and the increment of stimulus intensity was set to 1% MO instead of 10% MO to determine RMT.

In the study of Cermak et al., a headpost was implanted into rat skull serving as a fixation of the TMS coil and RMT under wakefulness was 34.6 ± 6.3% maximum stimulator output, which was estimated by observing limb/paw twitches [26]. However, the device proposed by Cermak et al. did not consider recording MEP in awake rats while performing TMS. Preimplantation of subcutaneous recording electrodes is a promising technique, which has been used to measure MEP induced by subcutaneous electrical stimulation in awake rats [35, 36]. In future research, a focused rodent TMS coil [18], an implanted headpost serving as a fixation of the TMS coil and the preimplantation of subcutaneous recording electrodes are promising techniques to measure MEP under wakefulness while performing TMS. However, in order to avoid obvious interference on MEP due to free movement of the limbs, the restraint is still necessary.

### Anesthesia inhibited corticospinal excitability

In the present study, the RMT under anesthesia was 55.6 ± 2.9% MO (Fig. 2A), and the peak-to-peak MEP amplitudes of 60% MO and 120% RMT were 258.6 ± 32.7 μV (Fig. 2C) and 326.1 ± 19.4 μV (Fig. 3C) respectively. Using the same magnetic stimulator and coil, our results were similar to that under urethane in the study by Sykes et al. [16]. Compared to that under wakefulness, the RMT under anesthesia increased by 11% MO (Fig. 2A) and the peak-to-peak MEP amplitudes of 60% MO decreased by 146 μV (Fig. 2C). A recent study by Gersner et al. demonstrated that the MEP amplitude in both low propofol bolus group (10 mg/kg) and high propofol bolus group (20 mg/kg) were significantly lower than the control group (no bolus) [27]. As anesthesia was maintained using continuous propofol infusion (1 mg/kg per min) before the experiment, it was not clear whether the rats were fully awake in the control group, nor were any measures taken to limit their movements [27]. Compared with the study by Gersner et al., the major highlight of this study is the explicit comparison between awake and anesthetized MEP. This study provided explicit evidence for the inhibition of anesthesia on corticospinal excitability. Moreover, the SR slope under anesthesia was significantly lower than that under wakefulness in this study (Fig. 3E). As the SR is collectively produced by multiple stimulation intensities, the SR slope is considered a more reliable and robust measure of corticospinal excitability [23, 37].

According to the recent study [21], isoflurane inhibited the excitatory transmitter release by blocking presynaptic Ca2 + channels and exocytic machinery. In addition, the protein level of α5 GABAA receptor (α5GABAAR), gephyrin, and dystrophin were significantly increased under isoflurane [37]. These factors may be the reasons that isoflurane inhibits corticospinal excitability. It is important to note that the reduced corticospinal excitability by isoflurane in this study refers to the excitability of the entire pathway from cortex to spine.

### The influence of anesthesia on rTMS-induced modulation

As shown in Fig. 4A and 5C, high-frequency rTMS facilitated corticospinal excitability in both awake and anesthetized conditions, with a reduced RMT and increased peak-to-peak MEP amplitudes after rTMS. The modulation of corticospinal excitability induced by 10 Hz rTMS was greater than that induced by 5 Hz rTMS. The stimulation frequency of rTMS has a significant influence on the efficacy of rTMS [38]. According previous studies, high-frequency rTMS produce after-effects via inducing long-term potentiation (LTP) on synaptic activity in a frequency-dependent manner [12, 38], which may be the reason why 10 Hz rTMS modulates corticospinal excitability more strongly than 5 Hz rTMS.

Under anesthesia, high-frequency rTMS still showed significant modulation of corticospinal excitability. Namely, even though the animals were anesthetized, neuromodulation was still visible. Several previous studies using rTMS under anesthesia improved neuroplasticity and restored memory impairment [39, 40]. Our results further confirmed the findings of rTMS under anesthesia. Moreover, our results showed that high frequency rTMS restored reduced corticospinal excitability to a certain extent, as shown in Fig. 5C. For human neurosurgery, anesthesia is indispensable and the corticospinal excitability is decreased during surgery. Alleviating the inhibition of corticospinal excitability by intraoperative high-frequency rTMS may be of great significance for the accurate identification of cortical function in surgery.

It’s worth noting that the increase in MEP of 110% RMT was significantly lower under anesthesia than under wakefulness, indicating the modulation of MEP was weaker than that under wakefulness. Studies have indicated that the baseline excitability state and spontaneous neural activity during stimulation can dramatically affect the modulation of rTMS [11, 23, 41]. However, the decrease in RMT between wakefulness and anesthesia was not significantly different, namely, anesthesia did not affect rTMS-induced modulation of RMT. RMT represents the threshold for generating MEP. In this study, the comparison of MEP amplitude was under a supramaximal stimulus intensity (110% RMT). A previous study revealed that the threshold for producing an MEP reflected the excitability of a central core of neurons that arises from the excitability of individual neurons and their local density [42]. The MEP induced by a supramaximal stimulus intensity may involve neurons that are intrinsically less excitable or spatially further from the center of activation [42]. Pharmaco-TMS-EMG studies strongly supports that MT represents cortico-cortical axon excitability, directly excited by TMS at threshold intensity with the induced current oriented along the anterior-to-posterior axis [43, 44]. MEP amplitude elicited by supramaximal stimulus intensity reflects transsynaptic activation of corticospinal neurons through a complex network of excitatory circuits controlled by inhibitory circuits [43, 44]. The RMT and MEP reflected corticospinal excitability from two levels of threshold stimulus intensity and suprthreshold stimulus intensity respectively. The specific mechanism underlying how anesthesia affects the rTMS-induced modulation of MEP still needs further study.

### Technical limitations

In the present study, latencies were shorter than that in some existing TMS-MEP studies, probably because we administered magnetic stimulation with higher intensity. According to three TMS-MEP studies [3, 6, 9], the latency of MEP decreased with the increase of the stimulus intensity, namely, the greater stimulus intensity would lead to a shorter latency. In five TMS-MEP studies [3, 6, 7, 14, 16], the threshold of MEP was 15 μV, 20 μV or 50 μV. In our study, to quickly identify MEP online, the threshold was set to 100 μV, which was much higher than that in previous five TMS-MEP studies. This meant that the absolute machine output corresponding to 100% MT in our study was stronger than that of the other four studies. As a result, latencies in our study were shorter.

Before rTMS, the RMT of all three groups were measured. After rTMS, the RMT was not remeasured again for the sham group, as it would be essentially unchanged over such a short time interval in the absence of effective stimulation. The study by Cermak et al. revealed no significant difference in MT across three days [26]. We acknowledge that this is a limitation of the study, although the main conclusions are not affected.

## Conclusions

In conclusion, the present study systematically measured TMS-induced MEP and rTMS-induced modulation and explicit comparisons were carried out between awake and anesthetized rats. Under anesthesia, corticospinal excitability was suppressed, but rTMS-induced modulation remained, although the modulation of MEP peak-to-peak amplitudes was weaker than that under wakefulness. This study provided explicit evidence for the inhibitory effects of anesthesia on corticospinal excitability and provided reference for the application of rTMS.

## Data Availability

The data is available from the corresponding author (Tao Yin) with reasonable request.

## Abbreviations

TMS: Transcranial magnetic stimulation
rTMS: Repetitive TMS
MEP: Motor evoked potential
RMT: Resting motor threshold
SR: Stimulus response
MO: Maximum output
